# Classification and Clinical Diagnosis of Fibromyalgia Syndrome: Recommendations of Recent Evidence-Based Interdisciplinary Guidelines

**DOI:** 10.1155/2013/528952

**Published:** 2013-11-26

**Authors:** Mary-Ann Fitzcharles, Yoram Shir, Jacob N. Ablin, Dan Buskila, Howard Amital, Peter Henningsen, Winfried Häuser

**Affiliations:** ^1^Division of Rheumatology, McGill University Health Centre, Alan Edwards Pain Management Unit, McGill University Health Centre, Canada H3G 1A4; ^2^Alan Edwards Pain Management Unit, McGill University Health Centre, Canada H3G 1A4; ^3^Department of Rheumatology, Tel Aviv Sourasky Medical Center, 64329 Tel Aviv, Israel; ^4^Department of Medicine, H. Soroka Medical Center, 84101 Beer-Sheva, Israel; ^5^Department of Medicine “B” and Centre for Autoimmune Diseases, Sheba Medical Centre, 52621 Tel Hashomer, Israel; ^6^Department Internal Medicine I, Klinikum Saarbrücken, Winterberg 1, 66119 Saarbrücken, Germany; ^7^Department of Psychosomatic Medicine and Psychotherapy, Technische Universität München, 81865 München, Germany

## Abstract

*Objectives*. Fibromyalgia syndrome (FMS), characterized by subjective complaints without physical or biomarker abnormality, courts controversy. Recommendations in recent guidelines addressing classification and diagnosis were examined for consistencies or differences. *Methods*. Systematic searches from January 2008 to February 2013 of the US-American National Guideline Clearing House, the Scottish Intercollegiate Guidelines Network, Guidelines International Network, and Medline for evidence-based guidelines for the management of FMS were conducted. *Results*. Three evidence-based interdisciplinary guidelines, independently developed in Canada, Germany, and Israel, recommended that FMS can be clinically diagnosed by a typical cluster of symptoms following a defined evaluation including history, physical examination, and selected laboratory tests, to exclude another somatic disease. Specialist referral is only recommended when some other physical or mental illness is reasonably suspected. The diagnosis can be based on the (modified) preliminary American College of Rheumatology (ACR) 2010 diagnostic criteria. *Discussion*. Guidelines from three continents showed remarkable consistency regarding the clinical concept of FMS, acknowledging that FMS is neither a distinct rheumatic nor mental disorder, but rather a cluster of symptoms, not explained by another somatic disease. While FMS remains an integral part of rheumatology, it is not an exclusive rheumatic condition and spans a broad range of medical disciplines.

## 1. Introduction

Roughly 2% of the developed world's population meet either the 1990 classification or 2010 modified diagnostic criteria of the American College of Rheumatology (ACR) for fibromyalgia syndrome (FMS) [1–5]. FMS patients report a wide array of somatic and psychological symptoms, with each contributing to a varying degree of symptom burden and functional disablement [6, 7]. FMS continues to present a challenge for healthcare professionals of various disciplines as well as for patients. Areas of contention include the benefits or harms of the diagnostic label “FMS,” the classification of the syndrome (rheumatic, neurologic, psychological disorder or a functional somatic syndrome), and the tender point examination that surfaced as a new physical finding two decades ago [8, 9]. The aim of the current review is to compare the recommendations of recent evidence-based interdisciplinary guidelines to identify consistencies but also to examine the presence of any contradictory conclusions regarding the definition (labelling) and clinical diagnosis of FMS.

## 2. Materials and Methods

### 2.1. Search of Literature

A systematic search of the US Agency for Healthcare Research and Quality (AHRQ) American National Guideline Clearing House (NGC) (http://www.guideline.gov/), the Scottish Intercollegiate Guidelines Network (SGN) (http://www.sign.ac.uk/guidelines/index.html), and the Guidelines International Network (G-I-N) (http://www.g-i-n.net/) was conducted from January 2008 to February 2013, using the key words “Fibromyalgia” and “Fibromyalgia Syndrome.” Medline was also searched from January 2008 to February 2013 with the search terms “Guideline” (publication type) and “Fibromyalgia” (mesh). Because regular updates of guidelines are required by guideline clearing houses, the searches were limited to this period. A manual search of the guideline bibliographies and contacts to international FMS key opinion leaders was undertaken to verify that all published guidelines were identified.

### 2.2. Inclusion Criteria

To be included in our analysis, the guidelines had to meet the following criteria.

1. The guideline was commissioned by a scientific organisation.
2. The guideline group was interdisciplinary and included at least the specialties rheumatology, pain medicine, and psychiatry or psychosomatic medicine or clinical psychology.
3. A systematic search strategy was outlined.
4. Recognized criteria of classification evidence and recommendations were used.
5. The formal process for establishing recommendations (Delphi exercise, panel conference) was outlined.

Guidelines that included FMS with other diagnoses, such as chronic fatigue syndrome, myalgic encephalomyelitis, or somatoform disorders, were excluded.

### 2.3. Analysis of the Guidelines

Inclusion criteria and the composition of the steering committees and panels, search strategies, the classification of evidence and recommendations, the procedures for establishing recommendations, and the recommendations given by the guidelines that met inclusion criteria were assessed by two independent reviewers (Mary Ann Fitzcharles, Winfried Häuser). All discrepancies were rechecked and consensus achieved by discussion. If needed a third reviewer was involved (Jacob N. Ablin).

## 3. Results

### 3.1. Guideline Selection

The literature search yielded 24 citations (1 in NGC, none in SIGN, 2 in GIN, and 21 in Medline). FMS opinion leaders reported two guidelines. Three of these met our inclusion criteria: the 2012 Canadian Guidelines for the diagnosis and management of fibromyalgia syndrome [10], the guidelines of the Association of the Scientific Medical Societies in Germany (AWMF) on the definition, pathophysiology, diagnosis, and treatment of fibromyalgia syndrome [11–19], and the Israeli guidelines for the diagnosis and treatment of fibromyalgia syndrome [20]. The reasons for excluding other hits were as follows: duplications (*n* = 19), not commissioned by a scientific society (*n* = 2) [21, 22].

### 3.2. Organisations Asked for the Development of the Guidelines

The Canadian guidelines were endorsed by the Canadian Pain Society (CPS) and the Canadian Rheumatology Association (CRA). The guidelines were developed by the Canadian Fibromyalgia Guidelines Committee (CFGC) [10].

The German guidelines were initiated and coordinated by the German Interdisciplinary Association of Pain Therapy (DIVS). The DIVS, an umbrella organisation of 18 scientific societies, is dedicated to the improvement of interdisciplinary pain therapy. The methodological development of the guidelines was supported by the Association of the Scientific Medical Societies (AWMF), the umbrella organisation of 152 scientific medical societies in Germany. Nine scientific associations (children and juvenile rheumatology, neurology, orthopedic surgery, pain medicine, pain psychology, physical therapy, and rehabilitation medicine, rheumatology, psychiatry, and psychosomatic medicine) and two national FMS self-help organisations participated in the guidelines development [11].

The Israeli guidelines were developed by the Israeli fibromyalgia group, on behalf of the Israeli Rheumatology Association. This group was formed within the national rheumatology association and included a group of experts with both clinical and research interest in FMS [20].

### 3.3. Composition of the Working Groups and Sources of Funding for the Guidelines

Details of the guideline groups are outlined in Table 1.

The development of the guidelines was funded by a private foundation (Canada), pharmaceutical companies (Canada, Israel), and the scientific societies involved (Germany, Israel). All three guideline groups comprised multidisciplinary teams representing healthcare professionals from relevant fields involved in the care of FM patients.

### 3.4. Methodologies

Details of the composition of the methodologies to design levels of evidence and grades of recommendations are outlined in Table 2.

The levels of evidence were assigned according to the classification system of the Oxford Centre for Evidence Based Medicine [23] by the Canadian and German guidelines [10, 11]. Both guidelines defined criteria for up- and downgrading the level of evidence. Grading of the strengths of recommendations was done according to the standards set out by the Oxford Centre for Evidence Based Medicine [23] for the Canadian guidelines and by the program for disease management guideline for the German guideline [24]. The Canadian guidelines were reviewed by an international expert and thereafter underwent external review requested by the Canadian Pain society using the AGREE II Score Sheet guideline appraisal tool [25]. The German guidelines were reviewed by three external reviewers of different medical specialties and by the boards of the scientific societies involved. The Israeli recommendations were formulated based on strength of evidence identified (see Table 3).

### 3.5. Need for a Guideline

All three countries justified the need for development of guidelines on the basis of the high prevalence of FMS and the association of reduced health-related quality of life of patients and high healthcare costs as well as controversies surrounding diagnosis and management [10, 11, 20]. The prevalence rates of FMS—assessed by different methodologies—were comparable between the three countries: Canada 2-3% [25], Germany 2.1% [5], and Israel 2.6% [3].

### 3.6. Recommendations for Definition and Classification

Recommendations concerning the definition, classification, clinical diagnosis, and general principles of care set out by all three guidelines were predominantly based on expert consensus, with very limited evidence available in the current literature.

All three guidelines defined FMS by the 1990 classification criteria of the American College of Rheumatology (ACR) classification criteria [1].

The Canadian guidelines stated that FMS is clinical construct of pain and other symptoms that cannot be explained by some other illness [10]. The German guidelines classified FMS as a functional somatic syndrome [13], defined by a typical cluster of symptoms and the exclusion of a somatic disease (e.g., endocrine or inflammatory) which sufficiently explains the symptoms [26]. The terms “FMS,” “somatoform pain disorder,” and “(masked) depression” are not interchangeable since not all patients with FMS meet the criteria of a somatoform pain disorder or a (masked) depression [13]. The Israeli guidelines [20] classified FMS to be a central hypersensitivity syndrome [27]. Both the Canadian and German guidelines identified FMS as a continuum disorder similar to other illnesses such as diabetes, rather than as a discrete disorder which could be present or absent at a particular time point. The prevalence of the syndrome depends on the cutoffs used for the definition of the disease/disorder, but is recognized as a condition that can wax and wane over time [10, 12].

### 3.7. Recommendations for Clinical Diagnosis

Details of the recommended diagnostic workup are outlined in Table 4.

Currently there is no specific diagnostic laboratory test or biomarker available for the diagnosis of FMS. All three guidelines were in agreement that the diagnosis remains clinical and the purpose of the physical examination and limited laboratory investigations is to rule out some other alternative diagnoses. The Canadian guidelines [10] recommended that the preliminary ACR 2010 diagnostic criteria may be used to validate a clinical diagnosis of FMS [28]. The German guidelines recommended [12] the use of the ACR 1990 classification criteria [1] or the modified ACR 2010 diagnostic criteria [4] or the AWMF criteria [29]. The Israeli guidelines did not make a specific recommendation regarding the use of 1990 or 2010 ACR criteria (which have yet to be formally adopted by the ACR).

The German guidelines recommended the use of the fibromyalgia survey questionnaire [30] to assess FMS-like symptoms and severity (see the supplementary Table in Supplementary Material online at http://dx.doi.org/10.1155/2013/528952).

Both the Canadian and the German guidelines stated that, for most patients, the diagnosis can be established by a primary care physician. A referral to a specialist (e.g., rheumatologist, neurologist, or endocrinologist) should be limited to situations when there is a reasonable clinical suspicion of some other condition that is presenting similarly to FMS or when the patient presents particular treatment challenges [10, 13].

All three guidelines emphasized that the diagnosis of FMS can coexist with a diagnosis of another somatic disease (e.g., inflammatory arthritis, osteoarthritis) or of a mental disorder (e.g., depression) [10, 13, 20]. The German guidelines recommended active screening for mental disorder and referral to mental healthcare specialist in case of suspected mental disorder or maladaptive coping [12, 14]. The Israeli guidelines recommended screening for symptoms of anxiety and depression as part of the initial evaluation [20]. The Canadian guidelines recommended that that the healthcare professional should be aware that psychological conditions may present with body pain [10].

### 3.8. Education after Initial Diagnosis of FMS

All three guidelines recommended that the diagnostic label “FM” or “FMS” should be communicated to patients after initial diagnosis and that patients should be provided with a clear explanation regarding the nature of the disorder, planned treatment strategy, and expected outcome [10, 11, 20]. This approach is intended to reduce anxiety, which inherently accompanies chronic pain [20]. There was also consensus that patients should be informed of the concept of a biopsychosocial model for FMS whereby biological factors (e.g., genetic predisposition) and psychosocial factors (e.g., stress) contribute to the predisposition, triggering, and perpetuating of FMS symptoms [10, 12, 20]. The Canadian guidelines discouraged excessive focus on a triggering event (such as a physical or psychological traumatic event) which could compromise patient care [10]. The German guidelines suggested that the following information could be useful in the education of patients.

- The symptoms are not caused by an organic disease (such as abnormality of muscles or joints) but are instead based on a functional disorder.
- The legitimacy of the ailment should be acknowledged.
- The symptoms are persistent in nearly all patients.
- Total relief of symptoms is seldom achieved.
- The symptoms do not lead to disablement and do not shorten life expectancy.
- Most patients learn to adapt to the symptoms over time.
- The goals of treatment are improvement in quality of life, maintenance of function (functional ability in everyday situations), and reduction of symptoms.
- The ability of the patient to modulate symptoms via self-management strategies should be emphasized [11].

The German guidelines group developed a patient version of the guideline and handouts for patients and their significant others, which should be distributed to the patient after establishing the diagnosis [11].

## 4. Discussion

We have identified considerable consistency between three recently published FMS guidelines spanning three continents. This agreement observed for the classification and clinical steps to establishing a diagnosis of FMS should put to rest many of the contentious issues that have challenged the medical community regarding this condition [8]. It should now be fully accepted that FMS is neither a distinct rheumatic disease nor a mental disorder, but a syndrome defined by a typical cluster of symptoms, with the exclusion of some other illness which sufficiently explains the symptoms. While FMS was originally defined by the ACR 1990 classification criteria [1], the cluster of symptoms which defines FMS goes beyond chronic widespread pain and tenderness. Physical and/or mental fatigue and sleep disturbance resulting in unrefreshed sleep are other key symptoms. Most patients report the presence of additional somatic and psychological symptoms. The existence of polysymptomatic distress, or symptoms beyond body pain, constitutes a “minor” diagnostic criterion of the preliminary ACR 2010 diagnostic criteria [28] and subsequent modification for survey and clinical use [4].

The diagnosis of FMS can be readily established in most cases by primary care physicians following a history of a typical cluster of symptoms and a defined diagnostic workup, including a complete medical history and physical examination and some simple and selected laboratory tests to exclude a somatic disease that sufficiently explains the symptoms. The modifier “sufficiently” in this definition is of critical importance and must be acknowledged: FMS often coexists with other disorders and the presence of such comorbidities must not be interpreted as ruling out a diagnosis of FMS. Thus, FMS is not a diagnosis of exclusion but rather a positive clinical diagnosis important to recognise, either independent of or in addition to other medical problems. In most patients diagnosed with FMS, more than one diagnosis is necessary to capture the whole spectrum of symptoms. Most importantly, other functional somatic syndromes (e.g., irritable bowel syndrome), mental disorders (e.g., depression or posttraumatic stress disorder), and somatic diseases (e.g., inactive or slightly active inflammatory rheumatic disease) can be diagnosed [12, 20].

A tender point examination is not obligatory for the diagnosis of FMS. This physical finding, subject to variable interpretation and which reflects an overall reduction in pain threshold, has at times been inappropriately used to establish a diagnosis of FMS. A referral to a specialist should be reserved for those selected patients in whom there is a suspicion of some other somatic disease and/or of mental disorder or when there are specific treatment challenges. Excessive healthcare utilization with referral to multiple specialists and repeated radiographic and laboratory investigations should be discouraged. The use of the (modified) ACR 2010 diagnostic criteria [4, 28], which do not require tender point examination, is recommended for clinical diagnosis, but should not preclude a thorough physical examination. While some patients with FMS will still be referred to rheumatologists, mostly to exclude some other rheumatic condition, FMS should no longer be identified as an exclusive rheumatic syndrome.

It is reassuring that the concordance rates of the different diagnostic criteria of FMS are high (80–90%) [29, 30]. This reinforces the true existence and validity of a condition that has caused so much consternation over the years. The concept of FMS, however, remains a work in progress with many current unanswered clinical and pathophysiologic questions. These recent guidelines as well as the revision of criteria for the diagnosis of FMS are clearly steps towards a better understanding of this condition. The ACR 2010 criteria will also likely lead to higher rates of FMS diagnosis in men because healthy [31] as well as men diagnosed with FMS [32] have less tender points than women.

## 5. Conclusions

FMS, often disputed and challenged, has emerged as a clinical syndrome with a clear cluster of symptoms and comorbidities. Despite the ongoing paucity of biomarkers available for diagnosing and monitoring of this condition, a systematic evidence-based approach can lead to effective, patient-centered management with avoidance of unnecessary and harmful interventions.

The cluster of symptoms that we today recognize as FMS has been described in the literature for over 200 years, with the specific diagnostic label of FMS introduced at the end of the 20th century [33]. The recent evidence-based interdisciplinary guidelines developed in Canada, Germany, and Israel should give healthcare professionals confidence to positively diagnose this condition, avoid excessive testing and medical consultation, and facilitate patient care by emphasis on appropriate patient education and active patient participation in healthcare plan.

## Supplementary Material

Fibromyalgia Survey Questionnaire (Polysymptomatic Distress Scale) contanis the symptom severity score and the widespread pain index

## Figures and Tables

**Table 1:** Comparison of the composition of the guideline groups and the funding of the Canadian, German, and Israeli guidelines.

	Canada	Germany	Israel
Nomination of members of the guideline group	Nominated by Canadian scientific societies	Nominated by German scientific societies or self-help organisations	Nominated by Israeli scientific societies or self-help organisations
Number of members in steering committee	11	12	11
Clinical expertise of members of steering committee			
Rheumatology/internal medicine	3	2	9
Pain therapy	1	1	0
Orthopedics/rehabilitation	1	2	0
Neurology	1	1	0
Pediatrics	0	1	0
Family medicine	1	0	1
Pharmacology	0	0	0
Psychiatry	0	1	1
Psychological pain therapy	1	1	0
Psychosomatic medicine	0	1	
Self-help organisations/patient representative	1	2	0
Physiatry/physiotherapy	1	0	
Others	1	0	
Competing interests of members of steering committee	9	10	9
Sources of financial support	Louise and Alan Edwards Foundation, Valeant	Scientific medical and psychological societies; self-help organisations	Pfizer; scientific medical and psychological societies

**Table 2:** Comparison of the methodology of the Canadian, German, and Israeli guidelines.

	Canada	Germany	Israel
Needs assessment	Structured consultation with 139 healthcare professionals from relevant disciplines	Structured consultation within the guideline group (50 persons building 8 working groups)	Structured consultation within the guideline group
Databases	EMBASE, MEDLINE, PSYCHINFO, PUBMED, and Cochrane Library	Medline, PsychINFO, SCOPUS, and Cochrane Library	Medline, Cochrane Library
Dates of search strategy	Until July 2010	Until December 2010	Until April 2012
Sources of evidence	Systematic reviews, meta-analyses, and clinical trials	Systematic reviews with meta-analyses of pain, fatigue, sleep problems, quality of life, and drop out for any reasons in randomised controlled trials conducted by guideline group; harms of therapies as reported in RCTs and in the literature	Systematic reviews, meta-analyses, and clinical trials
Sources of recommendations	Systematic reviews, meta-analyses, RCTs, panel consensus, and approval by ≥80% of 35 members of the National Fibromyalgia Guidelines Advisory Panel (NFGAP)	Systematic reviews with meta-analyses conducted by guideline group; structured consensus conference*	Systematic reviews, meta-analyses, and RCTs; panel consensus
Number of references in the guideline document	336	608	30
Classification of evidence	Oxford criteria	Oxford criteria	Oxford criteria
Classification of recommendations	Oxford criteria	German national guidelines	Recommendations based on strength of evidence
External review	One international expert Boards of both endorsing scientific societies	Boards of scientific societies involved	Chairman of Israel Rheumatology Society
Publication	In press	April 13, 2012	In press
Internet access	http://www.canadianpainsociety.ca/pdf/Fibromyalgia_Guidelines_2012.pdf	http://www.awmf.org/leitlinien/detail/ll/041-004.html	http://www.ima.org.il/Ima/FormStorage/Type7/clinical_68_fibrom.pdf

*Strong consensus: >95% of the participants consented; consensus: 75–95% of the participants consented; majority: 50–75% of the participants consented. A minority statement and an explanatory statement were possible.

RCT: randomized controlled trial.

**Table 3:** Comparison of the categorisation of evidence (treatment) and recommendations of the Canadian, German, and Israeli guidelines.

	Canada	Germany	Israel
Evidence level I	SR of randomised controlled trials or n-of-1 trial*	Ia-SR (with homogenity) of RCTs** Ib-individual RCT (with narrow confidence Interval)** IC-all or none**	SR of randomised controlled trials with large number of participants (over 1000)
Evidence level II	Randomized trial or (exceptionally) observational studies with dramatic effect*	IIa-SR (with homogeneity) of cohort studies** IIb-individual cohort study (including low quality RCT; e.g., <80% followup)** IIc-“Outcomes” research; Ecological studies**	SR of observational studies, cohort studies, or small randomized studies
Evidence level III	Nonrandomized controlled cohort/follow-up study*	IIIa-SR (with homogenity) of case-control studies** IIIB-individual case-control study**	Nonrandomized controlled studies
Evidence level IV	SR of case-control studies, historically controlled studies*	Case-series (and poor quality cohort and case-control studies)**	SR of case-control studies, open studies, and case reports
Evidence level V	Expert opinion	Expert opinion without explicit critical appraisal or based on physiology, bench research, or “first principles”**	Expert opinion
Recommendation strength A	Consistent level I studies	Directly based on evidence level I***	“Strong evidence”: based on level I evidence
Recommendation strength B	Consistent level 2 or 3 studies **or** extrapolations from level 1 studies	Directly based on evidence level II or extrapolated recommendation evidence level I***	“Medium evidence”: based on level II evidence
Recommendation strength C	Level 4 studies **or** extrapolations from level 2 or 3 studies	Directly based on evidence levels III, IV, and V***	“Weak evidence”: based on levels III-IV evidence
Recommendation strength D	Level 5 evidence **or** troublingly inconsistent or inconclusive studies of any level		
Panel consensus	Opinion supported by entire Canadian Fibromyalgia Guidelines Committee	Recommendation supported by majority of guideline group****	Recommendation supported by entire Israeli fibromyalgia group panel

RCT: randomised controlled trial; SR: systematic review or meta-analysis.

*Level may be graded down on the basis of study quality, imprecision, and indirectness, because of inconsistency between studies or because the absolute effect size is very small; level may be graded up if there is a large or very large effect size.

**Level may be graded down on the basis of study quantity (<4 RCTs of <200 patients), study quality (low study quality according to van Tulder score), low external validity (exclusion of patients with inflammatory rheumatic diseases and/or anxiety or depressive disorders), and evidence of publication bias.

***An up- or downgrading of recommendations is possible depending on the consistency of the results of the studies, the clinical relevance of the outcomes and effect sizes of the studies, the benefit-harm ratio, ethical considerations, patients' preferences, and the applicability of the therapies.

****The strength of consensus was classified as follows: strong consensus: consent of >95%, consensus: consent of 75–95%, majority consent: consent of 50–75%, and no consent: consent of <50% of the participants. A minority vote with a substantial rationale was possible.

**Table 4:** Comparison of the recommendations of the Canadian, German, and Israeli guidelines on the clinical diagnosis of FMS.

	Canada	Germany	Israel
History of a typical cluster of symptoms	Diffuse body pain that has been present for at least 3 months, with symptoms of fatigue, sleep disturbance, cognitive changes, mood disorder, and other somatic symptoms to variable degree	Chronic widespread pain and fatigue (physical and/or mental) and sleeping problems/unrefreshed sleep	Presence of pain in muscles, joints, connective tissues, various areas of the upper and lower limbs, neck, shoulders, and upper and lower back Typical symptoms of sleep disturbances, difficulty falling asleep, frequent awakening during the night, disturbed sleep patterns, and unrefreshing sleep Chronic fatigue complaints throughout the day Difficulties with concentration and memory

Exclusion	Other illness explaining the symptoms	Somatic disease sufficiently explaining the symptoms; the diagnosis of a mental disorder does not exclude the diagnosis of FMS	Other disorders explaining the symptoms have been ruled out. FMS may develop in coexistence with additional disorders, be they somatic, inflammatory, psychiatric, or otherwise

Recommended methods for exclusion of a somatic disease	Complete physical examination Full blood count, erythrocyte sedimentation rate (ESR), C-reactive protein (CRP), creatine phosphokinase (CPK), and thyroid stimulating hormone (TSH)	Obtaining history of pharmacological agents used Complete physical examination Complete blood count, C-reactive protein (CRP), serum calcium, creatine phosphokinase (CPK), and thyroid stimulating hormone (TSH)	Complete physical examination Complete blood count, renal function tests (creatinine and urea), serum calcium and phosphorous levels, liver function tests, creatine phosphokinase (CPK), erythrocyte sedimentation rate (ESR), C-reactive protein (CRP), thyroid stimulating hormone (TSH) and vitamin D

Further tests	Any additional laboratory or radiographic testing should depend on the clinical evaluation in an individual patient that may suggest some other medical condition	Only in case of clinical hints pointing at a somatic disease	At the discretion of the physician performing the evaluation, based on clinical hints pointing at a somatic disease. (low threshold for serological tests e.g., ANA and RF)

Tender point examination	Specific tender point examination is not required, but examination of soft tissues for generalized tenderness should be done	Facultative	No requirement to document number of tender points; however, assessment of tenderness recommended as part of physical examination

Screening for mental disorders	Mental disorder can be expected in three quarters of persons with FMS	Recommended	Recommended

Diagnostic criteria	American College of Rheumatology (ACR) 2010 preliminary diagnostic criteria	ACR 1990 classification criteria or ACR 2010 modified diagnostic criteria AWMF criteria	Clinical diagnosis, based on above evaluation
