# Supplementary material for: Classification and Clinical Diagnosis of Fibromyalgia Syndrome: Recommendations of Recent Evidence-Based Interdisciplinary Guidelines
Source: Evid Based Complement Alternat Med. 2013 Nov 26;2013:528952. doi: 10.1155/2013/528952 (PMC3860136; doi:10.1155/2013/528952)
Supplement: Supplementary file 1 — Fibromyalgia Survey Questionnaire (Polysymptomatic Distress Scale) contanis the symptom severity score and the widespread pain index [file 528952.f1.pdf]

Table 5: Fibromyalgia survey questionnaire [25]

Symptom severity score (SSS)

I. Using the following scale, indicate for each item the level of severity **over the past week** by checking the appropriate box.

0: No problem

1: Slight or mild problems; generally mild or intermittent

2: Moderate; considerable problems; often present and/or at a moderate level

3. Severe: continuous, life-disturbing problems

Fatigue	□ 0	□ 1	□ 2	□ 3
Trouble thinking or remembering	□ 0	□ 1	□ 2	□ 3
Waking up tired (unrefreshed)	□ 0	□ 1	□ 2	□ 3

II. During the **past 6 months** have you had any of the following symptoms?

Pain or cramps in lower abdomen:	□ Yes (1)	□No (0)
Depression:	□ Yes (1)	□No (0)
Headache :	□ Yes (1)	□No (0)

Widespread pain index (WPI)

Please indicate below if you have had pain or tenderness over the past 7 days in each of the areas listed below. Please make an X in the box if you have had pain or tenderness. Be sure to mark both right side and left side separately

□ Shoulder, left<br>□ Shoulder, right	□ Upper leg, left<br>□ Upper leg, right	□ Lower back<br>□ Upper back<br>□ Neck
□ Hip, left<br>□ Hip, right	□ Lower leg, left<br>□ Lower leg, right	
□ Upper am, left<br>□ Upper arm, right	□ Jaw, left<br>□ Jaw, right	□ No pain in any of these areas
□ Lower rarm, left<br>□ Lower arm, right	□ Chest<br>□ Abdomen	

IV. Overall, were the symptoms listed in I - III above generally present for at **least 3 months**? □ Yes □ No

The criteria of the symptoms of FMS are met if > 7 pain sites in the WPI and the SSS score is > 4 and IV is answered with “yes” [6].
